# Electroacupuncture Treatment Suppresses Transcription Factor IRF8 in Spinal Cord of Rats with Spared Nerve Injury

**DOI:** 10.1155/2020/1854363

**Published:** 2020-04-10

**Authors:** Ying Wang, Meng Xue, Yang-yang Xia, Qian Jiang, Zhi-hua Huang, Cheng Huang

**Affiliations:** ^1^Department of Physiology, Gannan Medical University, Ganzhou 341000, China; ^2^Pain Medicine Research Institute, Gannan Medical University, Ganzhou 341000, China

## Abstract

**Objective:**

Neuropathic pain with complex mechanisms has become a major public health problem that greatly impacts patients' quality of life. Therefore, novel and more effective strategies against neuropathic pain need further investigation. Electroacupuncture (EA) has an ameliorating effect on neuropathic pain following spared nerve injury (SNI), but the underlying mechanism remains to be fully clarified. Interferon regulatory factor 8 (IRF8), a critical transcription factor, was reported to be involved in the modulation of neuropathic pain. Here, we focused on exploring whether 2 Hz EA stimulation exerts an inhibitory action on spinal IRF8 in SNI rats.

**Methods:**

In this study, SNI rats were treated with 2 Hz EA once every other day for 21 days. Paw withdrawal threshold (PWT) was applied to determine the analgesic effect of 2 Hz EA on SNI rats. The spinal IRF8 and CX3CRl expressions were detected with qRT-PCR and western blot, and immunofluorescence staining was used to evaluate colocation of IRF8 or CX3CRl with microglial activation marker CD11b in the spinal cord.

**Results:**

It was found that SNI induced significant elevation of spinal IRF8 and CX3CRl mRNA and protein expression. Additionally, immunofluorescence results showed that SNI elicited the coexpression of IRF8 with CD11b, as well as CX3CRl with CD11b in the spinal cord. Meanwhile, 2 Hz EA treatment of SNI rats not only reduced IRF8 and CX3CRl mRNA and protein expression, but also reversed the coexpression of IRF8 or CX3CRl with CD11b in the spinal cord, along with an attenuation of SNI-evoked mechanical hypersensitivity.

**Conclusion:**

This experiment highlighted that 2 Hz EA can inhibit IRF8 expression and microglial activation in the spinal cord of SNI rats. Hence, targeting IRF8 may be a promising therapeutic strategy for 2 Hz EA treatment of neuropathic pain.

## 1. Introduction

Neuropathic pain resulting from peripheral nerve injury severely affects millions of individuals and causes a great burden to the health care [1]. In clinical practice, neuropathic pain is closely associated with hyperalgesia, allodynia, and spontaneous pain. Moreover, the mechanisms underlying neuropathic pain are well complicated [1]. It is evident that nerve injury induces the activation of microglia in the spinal cord [2, 3], and the activated microglia can evoke central sensitization and lead to neuropathic pain [4, 5]. Under pathological conditions, some transcription factors participate in the modulation of microglial activation [6]. Recent studies reported that interferon regulatory factor 8 (IRF8), a key member of transcription factors (IRF1–9) superfamily, is abundantly expressed on the spinal microglia after nerve injury and plays a crucial role in activating microglia [7, 8]. Furthermore, spinal IRF8 not only promotes microglial activation but also triggers proinflammatory cytokine production including IL-1*β* and chemokines and then elicits neuropathic pain [7]. Conversely, knockout of IRF8 mice are not sensitive to pain hypersensitivity induced by nerve injury [7]. These results suggested that spinal IRF8 contributed to the pathogenesis of neuropathic pain through regulating microglial activation.

Microglia have been confirmed to be a kind of immune cell in the central nervous system and play an essential role in neuroinflammation [9, 10]. Evidence showed a critical role of neuroinflammation in the pathogenesis of neuropathic pain [11]. Proinflammatory cytokines, chemokines, and their receptors play an important role in the induction of neuropathic pain [7]. The spinal microglia are activated by proinflammatory mediators and their cell-surface receptors following nerve injury [3, 11, 12]. Meanwhile, the activated microglia are regarded as a major source for proinflammatory cytokines, CX3 chemokine, and its receptor CX3CR1, which are involved in neuropathic pain [3, 6, 11]. Further investigation has demonstrated that suppression of both microglial activation and CX3CR1 expression results in the alleviation of neuropathic pain [12]. This implied that a crosstalk between microglial activation and CX3CR1 expression increase participated in the development of neuropathic pain.

Currently, neuropathic pain is still a very serious international public health problem [1]. Thus, further investigation for available and more effective treatments against neuropathic pain is greatly needed [13]. It is well known that electroacupuncture (EA), an alternative of traditional acupuncture, has been widely used in China and other oriental countries for the management of neuropathic pain with considerably fewer side effects [14–16], but the underlying mechanisms remain to be elucidated. Increasing evidence revealed that the inhibitory effect of EA stimulation is highly related to the modulation of neuroinflammation [17–19]. Our recent study demonstrated that 2 Hz EA alleviated SNI-induced neuropathic pain through blockade of microglial activation and proinflammatory cytokine IL-1*β* release in the spinal cord [20, 21]. Other study also reported that CX3CR1 knockout mice exhibit the reduction of inflammatory and neuropathic pain and a decrease of spinal microglial response [22]. Additionally, under inflammatory pain conditions, EA stimulation was greatly associated with attenuation of microglial activation and spinal CX3CR1 expression [22, 23]. It is based on the fact that spinal IRF8 evokes microglial activation and accelerates proinflammatory mediators release contributing to neuropathic pain development [7]. Thus, in this experiment, we hypothesized that 2 Hz EA treatment may inhibit transcription factor IRF8 in the spinal cord following SNI-induced neuropathic pain.

In the present study, we aim at exploring whether 2 Hz EA stimulation regulated IRF8 and CX3CRl expression in the spinal cord of SNI rats and subsequently influenced the coexpression of IRF8 with CD11b, as well as CX3CRl with CD11b in the spinal cord of SNI rats. Finding from this experiment may provide further insight into the mechanisms underlying 2 Hz EA in treating SNI-evoked neuropathic pain.

## 2. Materials and Methods

### 2.1. Experimental Animals

Healthy adult male Sprague Dawley (SD) rats (weighing 160–180 g, seven to eight weeks old) were obtained from Hunan SLAC Laboratory Animal Co., Ltd. (Changsha, P. R. China). Rats were housed five per cage with free access to food pellets and water, which were kept in a temperature-controlled (22°C–24°C) room with a 12/12-hour light/dark cycle. All animal experimental procedures were approved by the Animal Use and Protection Committee of Gannan Medical University in China and carried out according to the guidelines of the International Association for the Study of Pain. All rats were acclimated to the environment for 5 days before this experiment. This study was performed in a quiet environment and conducted in a double-blind way. Every possible measure was performed to minimize pain to rats.

### 2.2. Experimental Procedure

**Figure 1:**
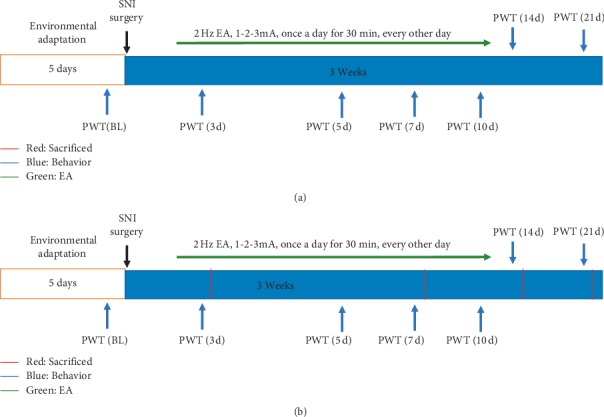
The schematic diagram of the experimental procedures. (a) The PWT was assessed following 2 Hz EA stimulation on days 3, 5, 7, 10, 14, and 21 postSNI surgery. Rats were given 2 Hz EA treatment with once every other day lasting for 21 days. (b) After 2 Hz EA treatment with once every other day for 21 days, the PWT of rat was measured on days 3, 7, 14, and 21 post-SNI surgery, and the samples were collected from the L4-L6 segments of rat spinal cord for immunofluorescence staining, qRT-PCR, and western blot experiments, respectively.

These experimental procedures were performed to define the molecular mechanism of 2 Hz EA in the spinal cord following SNI, and the protocols are shown in Figure 1. In the first procedure, rats were randomly divided into the sham group, the SNI group, and the SNI + EA group (*n* = 12 per group). Rats in the sham group obtained sham surgery, while the other groups received SNI surgery. 2 Hz EA treatment of rats in the SNI + EA group occurred once every other day for 21 days from day 1 post-SNI surgery. The rats in the sham and SNI groups did not receive EA stimulation. The paw withdrawal threshold (PWT) was evaluated before and after the administration of 2 Hz EA on days 3, 5, 7, 10, 14, and 21 post-SNI surgery, respectively. In the second procedure, further experiments investigated the mechanisms underlying 2 Hz EA attenuating SNI-induced neuropathic pain in the spinal cord. Rats from the sham group, the SNI group, and the SNI + EA group (*n* = 3–5 per group) were used to harvest the L4-L6 segments of the spinal cord on days 3, 7, 14, and 21 postsurgery, respectively. The spinal cord at L4-L6 segments was processed for qRT-PCR, western blot, and immunofluorescence staining. A total of 50 rats were used in this research.

### 2.3. Establishment of Spared Nerve Injury Rat Model

After rats were adapted to the surrounding for 5 days, the SNI rat model of neuropathic pain was established as described in a previous report [24]. Briefly, rats were deeply anesthetized by 2% to 3% isoflurane, and then the left lateral sciatic nerve and its trifurcations (three terminal branches including sural, common peroneal, and tibial nerves) were exposed. The left common peroneal and tibial nerves were tightly ligated with a 5-0 silk thread, being sectioned distal to the ligation with the removal of 2–4 mm of the distal nerve stump, and keeping the sural nerve intact. The skin and muscle of rats were closed under sterile conditions. Only rats that developed pain hypersensitivity were used in this experiment. The mechanical hypersensitivity was examined by PWT as described in previous data [16]. The sham operation was performed in rats with all identical surgery protocols except that the common peroneal and tibial nerves were kept intact.

### 2.4. Assessment of Mechanical Hypersensitivity

Mechanical hypersensitivity was regarded as a behavioral sign of neuropathic pain. The sensitivity to mechanical stimulation was examined by PWT, as described in our previous study [20]. Briefly, rats were first acclimatized in individual plastic enclosures (12 × 22 × 18 cm) on a metal mesh floor standing for 15 min before testing. The PWT was detected by application of a dynamic plantar aesthesiometer (Ugo Basile, 37450, Italy), which comprises a force transduction fitted with a 0.5 mm diameter polypropylene rigid tip. A probe was applied perpendicularly to the midplantar surface of the rat left hind paw with increasing pressure, spending 10 s from 0 to 50 g. To avoid injury to the rat, the cutoff pressure was set to be 50 g; the force that induced the withdrawal response was automatically recorded to the nearest 0.1 g by the anesthesiometer. Three measurements were taken with an interval of 5 min. The mean PWT for three examinations was obtained for each rat.

### 2.5. Electroacupuncture Stimulation

2 Hz EA treatment of SNI rats was conducted as previously described [20, 21]. Briefly, rat was housed using a specially designed holder with its both hind legs and tail exposed; the skin of hind legs in the rat was sterilized using 75% alcohol. In some sites of each hind leg from rat, two stainless steel needles (4 mm length and 0.4 mm diameter) were inserted. One needle was inserted into Zusanli acupoint (ST36), which was 5 mm lateral to the anterior tubercle of the tibia marked by a notch, and the other needle was inserted into Sanyinjiao acupoint (SP6), which was 3 mm proximal to the medial malleolus and localized in the posterior border of the tibia. The square waves of EA stimulus were generated from Han's Acupoint Nerve Stimulator (HANS, LH202, Huawei Industrial Developing Company, Beijing, P. R. China), which were used to stimulate the hind legs of the rat simultaneously. In this experiment, EA stimulation frequency was used for 2 Hz with 0.6 ms pulse width, EA stimulus intensity was elevated in a stepwise way at 1-2-3 mA, and each stimulation intensity lasted for 10 min. 2 Hz EA treatment of rats occurred once every other day lasting for 21 days. During the process of EA stimulation, rats always were awakened.

### 2.6. Quantitative RT-PCR Analysis

IRF8 and CX3CRl mRNA expressions were detected with quantitative reverse transcription and real-time polymerase chain reaction (qRT-PCR). Following deep anesthesia with 2% to 3% isoflurane, the fresh ipsilateral lumbar (L4-L6) dorsal spinal cord segments of the rat were rapidly collected in a 1.5 ml centrifuge tube and stored in liquid nitrogen. Total RNA was isolated from the spinal cord segments at L4-L6 and prepared for qRT-PCR as described in the other study [16]. The primer sequences for qRT-PCR were provided by Invitrogen (USA); the primer sequences used in this experiment were as follows: IRF8: sense CCAGATCCTCCCTGACTGGT and antisense TAAGGCTGAATGGTGTGCGT; CX3CRl: sense TCGTCCTGCCCTTGCTTATC and antisense AGGCGTCCAGAAGAGGAAG; and GAPDH: sense CAGCCGCATCTTCTTGTGC and antisense GGTAACCAGGCGTCCGATA. Quantitative RT-PCR data were normalized with GAPDH mRNA level which was used as a control, and relative mRNA levels were expressed as 2^−∆∆Ct^ values.

### 2.7. Western Blot Analysis

Western blot assay was performed after the behavioral experiment. Under deep anesthesia with 2% to 3% isoflurane, the rat spine was quickly opened to expose the spinal cord, the ipsilateral spinal cord segments at L4-L6 were rapidly removed for the extraction of protein. This procedure was performed on ice. Thirty micrograms of protein per sample was denatured and then separated with 10% SDS-PAGE and western-blotted on a PVDF (Millipore, CA) membrane using a minigel and mini transblot apparatus (Bio-Rad, Hercules, CA). The membrane was blocked with 5% nonfat milk in Tris-buffered saline containing 0.1% Tween-20 for 60 min at room temperature, and subsequently, the membrane was respectively immunolabelled overnight at 4°C with antibodies of rabbit anti-IRF8 (1 : 400, ab28696, UK) or anti-CX3CR1 (1 : 300, ab8021, Abcam, UK), or *β*-tubulin (1 : 1000, Solarbio, China). The blots were washed with Tris-buffered saline and Tween and then incubated with the horseradish peroxidase-conjugated anti-rabbit secondary antibody (1 : 1000, Cell Signaling Technology, USA) for 60 min at 4°C. The blots site of the antigen-antibody complex was visualized with Immobilon Western Chemiluminescent HRP Substrate (Millipore, MA). The bands were analyzed using Quantity One software (Bio-Rad). *β*-Tubulin was used as internal control. The standardized ratio of IRF8 or CX3CRl protein to *β*-tubulin band density was used to calculate the alteration of the corresponding protein expression level.

### 2.8. Immunofluorescence Staining

After the behavioral assessment, rats were anesthetized with 2% to 3% isoflurane and transcardially perfused through the thoracic cavity, which was incised to expose the heart, and a puncture needle was inserted from the left apex and extended to the aorta with haemostatic forceps. The right auricle was incised and transcardially perfused with 0.9% normal saline and then 4% paraformaldehyde solution in 0.1 M phosphate-buffered saline (PBS, pH 7.4, 4°C). Immunofluorescence staining was performed as described in a previous study [25]. Briefly, the spinal cord segments at L4-L6 were immediately removed and put into 4% paraformaldehyde solution for 4 hours, followed by cryoprotectant in 20% and 30% sucrose, respectively, at 4°C until submerge. Transverse sections (10 *μ*m) were cut on a cryostat and blocked with 5% BSA for 60 min at room temperature, and after being washed with PBS for 15 min and incubated with 5% BSA for overnight at 4°C, the sections were incubated with anti-IRF8 (1 : 100, ab28696, UK) or anti-CX3CR1 (1 : 100, ab8021, Abcam, UK), respectively, and mouse anti-CD11b for microglia (1 : 400; ab1211, UK). After washing with PBS for 15 min, the membranes were incubated for 60 min at room temperature with Alexa Fluor TM 488-conjugated goat anti-rabbit IgG (1 : 1000; Molecular Probes, Carlsbad, CA) and Alexa Fluor TM 594-conjugated goat anti-mouse IgG (1 : 1000; Molecular Probes). All stained sections were detected and analyzed with a confocal laser scanning fluorescence microscope.

### 2.9. Statistical Analysis

Statistical analyses were performed using Prism 5.0 software. The differences between groups in behavioral experiments, qRT-PCR, and western blot were analyzed using two-way repeated-measures ANOVA followed by Bonferroni's post hoc tests. All experimental data were expressed as the mean ± SEM. *P* < 0.05 was considered statistically significant.

## 3. Results

### 3.1. Effect of 2 Hz EA on SNI-Induced Mechanical Hypersensitivity

**Figure 2:**
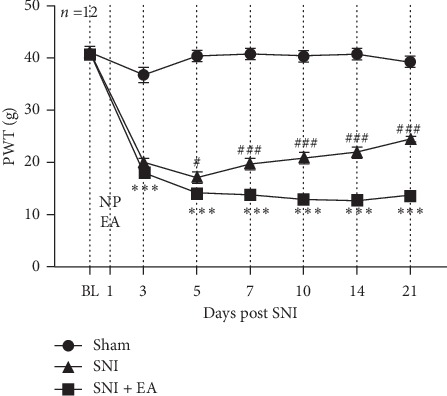
Effect of 2 Hz EA on mechanical paw withdrawal threshold evoked by SNI. Rats were treated with 2 Hz EA stimulation once every other day for 21 days postsurgery. Sham represents the sham group; SNI represents rats treated with the spared nerve injury-induced neuropathic pain; SNI + EA represents rats receiving spared nerve injury and 2 Hz EA treatment. ^*∗∗∗*^*P* < 0.001, compared to the sham group; ^###^*P* < 0.001, compared to the SNI group. All data were expressed as the mean ± SEM, *n* = 12 per group. Two-way repeated-measures ANOVA followed by Bonferroni's post hoc test.

To investigate the effect of 2 Hz EA treatment on mechanical hypersensitivity induced by SNI, SNI rats were treated with 2 Hz EA once every other day lasting for 21 days postsurgery, and the PWT was measured. The results are shown in Figure 2; in comparison with the sham group, the PWT was significantly decreased in the SNI group (*P* < 0.001), which suggested that the rat model of SNI-induced neuropathic pain was successfully established. However, compared with the SNI group, the PWT was obviously enhanced in the SNI + EA group on days 3, 5, 7, 10, 14, and 21 postsurgery, respectively (*P* < 0.05, *P* < 0.001), indicating that 2 Hz EA stimulation can attenuate mechanical hypersensitivity induced by SNI.

### 3.2. Effect of 2 Hz EA on Spinal IRF8 mRNA and Protein Expression following SNI

**Figure 3:**
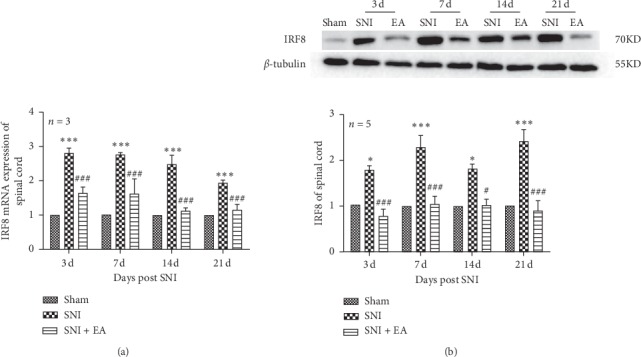
Effect of 2 Hz EA on spinal IRF8 expression in SNI rats. (a) Relative level of IRF8 mRNA expression. (b) Western blotted band of IRF8 and relative level of IRF8 protein expression. Samples were collected from the L4-L6 segments of the spinal cord on days 3, 7, 14, and 21 postsurgery. ^*∗*^*P* < 0.05, ^*∗∗∗*^*P* < 0.001, compared to the sham group; ^#^*P* < 0.05, ^###^*P* < 0.001, compared to the SNI group. All data were expressed as the mean ± SEM, *n* = 3–5 per group. Two-way repeated-measures ANOVA followed by Bonferroni's post hoc tests.

To assess the effect of 2 Hz EA stimulation on spinal IRF8 in SNI rats, the expression levels of spinal IRF8 were detected on days 3, 7, 14, and 21 post-SNI surgery. As shown in Figure 3, in the qRT-PCR experiment, SNI rats have significant higher IRF8 mRNA expression level in the spinal cord than rats in the sham group (*P* < 0.001) on days 3, 7, 14, and 21 postsurgery, respectively, whereas, 2 Hz EA treatment distinctly reduced spinal IRF8 mRNA expression (*P* < 0.001) on days 3, 7, 14, and 21 postsurgery, respectively. Furthermore, IRF8 protein expression was obviously upregulated in the spinal cord of SNI rats compared to that in the sham group (*P* < 0.05, *P* < 0.001) on days 3, 7, 14, and 21 postsurgery, respectively. However, 2 Hz EA treatment of SNI rats markedly decreased spinal IRF8 protein expression (*P* < 0.05, *P* < 0.001) on days 3, 7, 14, and 21 postsurgery, respectively. These results revealed that 2 Hz EA can decrease the elevation of IRF8 expression induced by SNI in the spinal cord.

### 3.3. Effect of 2 Hz EA on Coexpression of Spinal IRF8 with CD11b in SNI Rats

**Figure 4:**
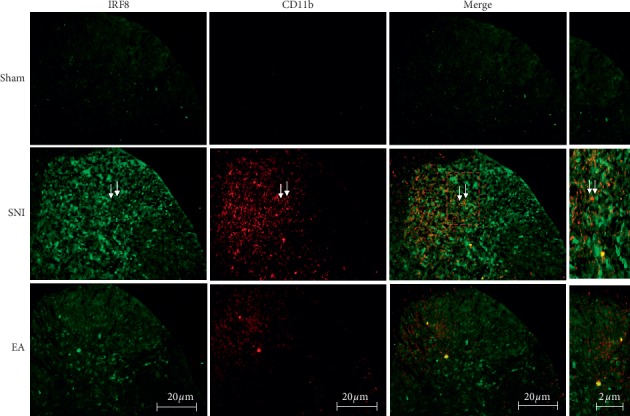
Immunofluorescence staining detected the effect of 2 Hz EA on coexpression of spinal IRF8 with CD11b in SNI rats. IRF8 and CD11b were double-stained in the spinal dorsal horn from the sham, SNI, and SNI + EA group rats (scale bar = 20 *μ*m). Immunofluorescence staining results showed that SNI enhanced spinal IRF8 and CD11b expression compared with the sham group. 2 Hz EA treatment reversed the coexpression of IRF8 (green) with microglial activation indicator CD11b (red) in the spinal cord of SNI rats.

To determine the potential role of 2 Hz EA on the relationship between IRF8 and microglial activation in the spinal cord of SNI rats, the effect of 2 Hz EA stimulation on the coexpression of IRF8 with the microglial activation marker CD11b in the spinal cord was detected by immunofluorescence staining, after day 7 postsurgery. As shown in Figure 4, compared with rats in the sham group, it was found that SNI increased spinal IRF8 expression; however, this effect was suppressed by 2 Hz EA treatment of SNI rats. Similarly, in comparison with rats in the SNI group, 2 Hz EA stimulation also inhibited the improvement of CD11b expression induced by SNI. Compared with rats in the sham group, we further found that SNI elicited the coexpression of IRF8 with CD11b in the spinal cord with double immunofluorescence staining. In contrast, the coexpression of spinal IRF8 with CD11b induced by SNI was reversed by 2 Hz EA treatment of SNI rats.

### 3.4. Effect of 2 Hz EA on Spinal CX3CRl mRNA and Protein Expression following SNI

**Figure 5:**
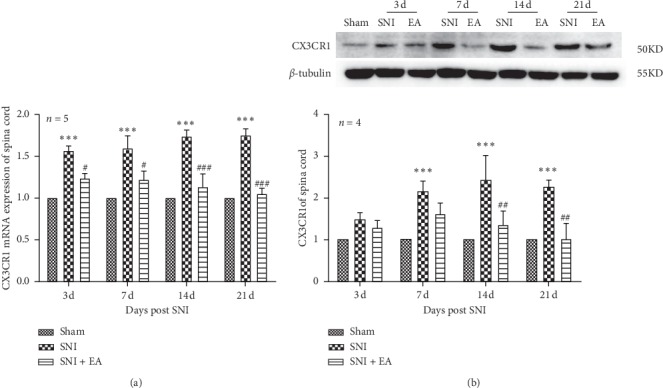
Effect of 2 Hz EA on spinal CX3CRl expression in SNI rats. (a) Relative level of CX3CRl mRNA expression. (b) Western blotted band of CX3CRl and relative level of CX3CRl protein expression. Samples were collected from the L4-L6 segments of the spinal cord on days 3, 7, 14, and 21 postsurgery. ^*∗∗*^*P* < 0.01, ^*∗∗∗*^*P* < 0.001, compared to the sham group; ^#^*P* < 0.05, ^##^*P* < 0.01, ^###^*P* < 0.001, compared to the SNI group. All data were expressed as the mean ± SEM, *n* = 4-5 per group. Two-way repeated-measures ANOVA followed by Bonferroni's post hoc tests.

To explore the effect of 2 Hz EA treatment on spinal CX3CRl in SNI rats, the mRNA and protein expression levels of CX3CRl in the spinal cord were determined on days 3, 7, 14, and 21 postsurgery. As shown in Figure 5, in comparison with the sham group, CX3CRl mRNA level in the SNI group was significantly upregulated (*P* < 0.001) on days 3, 7, 14, and 21 postsurgery, respectively, whereas, compared to the SNI group, CX3CRl mRNA expression in the SNI + EA group was obviously downregulated (*P* < 0.05; *P* < 0.001) on days 3, 7, 14, and 21postsurgery, respectively. Similarly, compared with the sham group, CX3CRl protein expression in the SNI group was markedly increased (*P* < 0.01, *P* < 0.001) on days 7, 14, and 21 postsurgery, respectively, while compared with the SNI group, 2 Hz EA treatment of SNI rats significantly decreased CX3CRl protein expression (*P* < 0.01) on days 14 and 21 postsurgery, respectively. These data showed that 2 Hz EA can suppress CX3CRl expression in the spinal cord induced by SNI.

### 3.5. Effect of 2 Hz EA on Coexpression of Spinal CX3CRl with CD11b in SNI Rats

**Figure 6:**
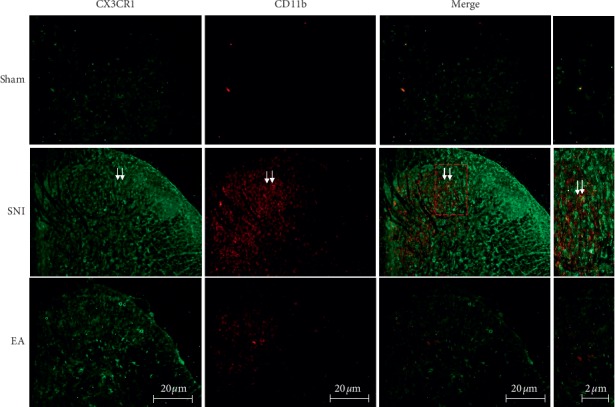
Immunofluorescence staining evaluated the effect of 2 Hz EA on coexpression of spinal CX3CRl with CD11b following SNI. CX3CRl and CD11b were double-stained in the spinal dorsal horn from the sham, SNI, and SNI + EA group rats (scale bar = 20 *μ*m). Immunofluorescence staining data revealed that the SNI improved spinal CX3CRl and CD11b expression compared to the sham group. 2 Hz EA treatment reversed the coexpression of CX3CRl (green) with microglial activation indicator CD11b (red) in the spinal cord of SNI rats.

To explore the possible effect of 2 Hz EA on the coexpression of CX3CRl with CD11b in the spinal cord of SNI rats, we used the microglial activation marker CD11b to assess the activation of microglia in the spinal cord, following day 7 postoperation. We also examined the coexpression of spinal CX3CRl with CD11b induced by 2 Hz EA stimulation with immunofluorescence staining. The results are shown in Figure 6. SNI induced the enhancement of spinal CX3CRl expression compared to rats in the sham group. However, in comparison with rats in the SNI group, 2 Hz EA treatment of SNI rats reduced spinal CX3CRl expression. Similarly, 2 Hz EA also inhibited the enhancement of CD11b expression induced by SNI. Double immunofluorescence staining results further demonstrated that SNI produced the coexpression of spinal CX3CRl with CD11b in the spinal cord, whereas 2 Hz EA treatment of SNI rats reversed the coexpression of CX3CRl with CD11b in the spinal cord.

## 4. Discussion

Neuropathic pain following nerve injury seriously impacts the life quality of patients [1]. An effective available management against neuropathic pain is still lacking. It was known that EA stimulation has an inhibitory effect on neuropathic pain [15, 16]. Novel molecular therapeutic targets for 2 Hz EA treatment of neuropathic pain need to be further identified. In this study, we found that SNI obviously elicited an upregulation of IRF8 and CX3CRl mRNA and protein expression in the spinal cord. Meanwhile, 2 Hz EA treatment of SNI rats can reduce IRF8 and CX3CRl expression and inhibit coexpression of IRF8 or CX3CRl with CD11b in the spinal cord. These findings expanded our understanding of molecular mechanisms underlying 2 Hz EA in relieving SNI-induced neuropathic pain.

In this experiment, the SNI rats exhibited obvious mechanical hypersensitivity, indicating that the SNI animal model was successfully established. Moreover, we found that the PWT of SNI rats is distinctly decreased on day 3 after SNI surgery, while the PWT on day 7 following SNI retains very low level, which is consistent with our previous report [20]. Based on the above evidence, the present research explored the activation of microglia starting from day 7 after SNI. This provides a stable animal model for further investigating the effect of EA stimulation on SNI-induced neuropathic pain. It is evident that EA treatment producing analgesic effect is greatly dependent on EA stimulation frequencies and EA stimulation intervals [26, 27]. Numerous studies demonstrated that the relieving effect of neuropathic pain induced by 2 Hz EA stimulation is better than that in 100 Hz EA [26, 27]. In addition, repeated or prolonged EA stimulation can induce EA tolerance [27]. Therefore, in this experiment, we chosen 2 Hz EA treatment of neuropathic pain with once every other day to avoid EA tolerance, as described in our previous report [20]. According to the other data [13, 16], after the administration of 2 Hz EA stimulating the acupoints of Zusanli and Sanyinjiao of SNI rats, the PWT significantly increased, suggesting that 2 Hz EA can effectively alleviate mechanical hypersensitivity in SNI rats. However, the mechanisms underlying the analgesic effect of 2 Hz EA on SNI-induced neuropathic pain need further research.

Neuroinflammation was reported to exert a critical effect on neuropathic pain [28–30]. Peripheral nerve injury elicits a neuroinflammatory response, including activation of microglia, secretion of proinflammatory cytokines, and alterations of pain-related gene expression that act as vital factors in neuropathic pain [3]. The activated spinal microglia were shown to play a crucial role in the modulation of neuropathic pain [22]. It is well established that IRF8 implicates in neuropathic pain through activating microglia and promoting proinflammatory cytokine and chemokine production in the spinal cord [7]. For example, IRF8 is specifically expressed on the spinal microglia and participates in regulating gene expression [7, 31]. The overexpression of IRF8 in cultured microglia facilitates the transcription of genes associated with reactive states, while the loss of IRF8 elicits the suppression of microglial activation in the spinal cord [7, 31]. It was found that IRF8 expression was significantly upregulated from day 1, peaked on day 3, and the enhancement of IRF8 lasted for several weeks following peripheral nerve injury [7]. Furthermore, the increased spinal IRF8 expression is closely linked to the activation of microglia in neuropathic pain [7, 32, 33]. These results indicated that both IRF8 in the spinal cord and its triggering microglial activation contributed to neuropathic pain development. In support of this notion, the current research further found that SNI obviously improved the levels of IRF8 mRNA and protein expression in the spinal cord from day 3 post-SNI surgery persisting for 21 days, and immunofluorescence results also manifested that IRF8 expression in the spinal cord was present at higher level in SNI rats. In addition, double immunofluorescence staining further demonstrated that, in comparison with rats from the sham group, IRF8 was coexpressed with microglial activation marker CD11b in the spinal cord of SNI rats. This strongly suggested that IRF8 was also well expressed on the spinal microglia in SNI rats, and SNI elicited the activation of microglia in the spinal cord, and this is in agreement with the previous studies [3, 7, 31, 32]. Hence, this experiment further confirmed that the upregulation of spinal IRF8 induced by SNI may play a crucial role in activating microglia, and its interaction with microglial activation led to SNI-induced neuropathic pain.

Owing to spinal IRF8 has emerged as an important initiator of neuropathic pain [7], we further explore whether 2 Hz EA relieved neuropathic pain via blockade of IRF8 signaling. Our recent study has reported that 2 Hz EA treatment of SNI rats can inhibit microglial activation in the spinal cord and alleviate pain hypersensitivity [20]. Similarly, in this experiment, we found that 2 Hz EA successfully attenuated the established mechanical hypersensitivity and produced remarkable downregulation of IRF8 mRNA and protein expression in the spinal cord of SNI rats. Double immunofluorescence staining further exhibited that the coexpression of IRF8 with CD11b induced by SNI was reversed by 2 Hz EA stimulation in the spinal cord. Therefore, our investigation suggested that suppressing spinal IRF8 and its activating microglia is likely to a potential approach for 2 Hz EA treatment of SNI-induced neuropathic pain.

CX3CL1 was reported to be critically involved in neuroinflammation [34], and its receptor CX3CR1 was mainly expressed on microglia [35]. It was clear that the interaction of CX3CL1 and CX3CR1 in the spinal cord plays a key role in inflammatory response and neuropathic pain initiation [36]. There is abundant evidence that CX3 chemokine and its receptor CX3CR1 were upregulated after peripheral nerve injury and contributed to neuropathic pain through its interaction with microglial activation in the spinal cord [30, 35]. It was found that SNL can markedly promote the elevation of CX3CR1 in the spinal microglia [35]. Conversely, intrathecal injection of an antibody against CX3CR1 in the spinal cord distinctly blocked mechanical allodynia induced by SNL [33]. Additionally, in CX3CR1 knockout mice, the spinal microglial activation was attenuated, the mechanical allodynia was not developed, and the thermal hypersensitivity was delayed after SNI [36]. The increase of CX3CR1 expression in spinal microglia following SNL has also been observed from OX-42, which is an indicator of microglial activation [37]. Collectively, these data implied that the interaction of CX3CR1 and microglial activation participated in the development of neuropathic pain.

Emerging evidence revealed that the analgesic effect of EA stimulation on neuropathic pain is highly related to immunoregulation [17, 18]. Moreover, proinflammatory cytokines and some signaling molecules participated in EA-induced analgesia in the spinal cord [38, 39]. In the current research, we observed a significant improvement of spinal CX3CR1 mRNA and protein expression following SNI. In addition, double immunofluorescence staining showed that CX3CR1 was colocalized with CD11b in the spinal cord of SNI rats, indicating SNI-evoked spinal microglial activation, and this is in parallel to the other data. Our experiment suggested that the enhancement of CX3CR1 expression and its triggering microglial activation in the spinal cord may be involved in the formation of SNI-induced neuropathic pain. By contrast, the upregulation of spinal CX3CR1 mRNA and protein expression induced by SNI was obviously decreased by 2 Hz EA treatment. Moreover, the double immunofluorescence studies demonstrated that 2 Hz EA stimulation can reverse the coexpression of CX3CR1 with CD11b in the spinal cord of SNI rats. Based on the evidence that microglia is the primary immune cell in the spinal cord [9], the activated microglia promotes proinflammatory mediator release in neuropathic pain [3, 6], while EA stimulation has the attenuating effect on neuropathic pain via immunomodulation [17]. Thus, our results strongly implied the involvement of neuroimmune effect of 2 Hz EA in alleviating SNI-induced neuropathic pain, such as inhibition of both CX3CR1 and microglial activation in the spinal cord of SNI rats by 2 Hz EA treatment.

## 5. Conclusion

In summary, the present experiment demonstrated that the upregulation of IRF8 and CX3CR1 expressions as well as microglial activation in the spinal cord contributed to SNI-induced neuropathic pain. Meanwhile, 2 Hz EA stimulation indeed blocked spinal IRF8 and CX3CR1 and the activation of microglia in SNI rats. Although further studies are needed to define the correlation of IRF8 or CX3CRl with microglial activation in the spinal cord following SNI by 2 Hz EA with inhibitors, our study provides a novel insight into the possible mechanism of 2 Hz EA in ameliorating SNI-induced neuropathic pain and suggests that targeting IRF8 might be a potential mechanism of 2 Hz EA against neuropathic pain.

## Data Availability

The data used to support the findings of this study are included within the article.
